# Hospitalization after Initial Telemedicine Versus in-Person Consultation for Outpatients with Respiratory or Digestive Diseases: A Retrospective Cohort Study

**DOI:** 10.31662/jmaj.2025-0245

**Published:** 2025-11-21

**Authors:** Takahito Morita, Yusuke Sasabuchi, Hideo Yasunaga

**Affiliations:** 1 Department of Clinical Epidemiology and Health Economics, Graduate School of Medicine, The University of Tokyo, Bunkyo-ku, Tokyo, Japan; 2 Department of Real-world Evidence, Graduate School of Medicine, The University of Tokyo, Bunkyo-ku, Tokyo, Japan

**Keywords:** telemedicine, initial consultation, hospitalization, Japan

## Abstract

**Introduction:**

We compared the proportion of hospitalizations in patients who received initial telemedicine consultations and in those who received in-person consultations.

**Methods:**

We used the DeSC database, a large administrative claims database for Japan, from April 2020 to November 2022. In this retrospective cohort study, we identified outpatients with respiratory or digestive diseases. The exposure group comprised patients who received an initial telemedicine consultation through the use of telephones and other telecommunication devices. The control group comprised patients who underwent an initial in-person consultation. The outcome measure was hospitalization within 1 month of the index date. Propensity score matching and multilevel logistic regression were performed, with patients at the subject level and medical institutions at the cluster level. A total of 3,026,260 eligible patients were identified. The index date for each patient was defined as the day when the patient was diagnosed with respiratory or digestive diseases at the initial consultation. Respiratory and digestive diseases were defined according to the International Classification of Diseases, 10th Revision codes.

**Results:**

The proportions of hospitalization within 1 month of the index date were 1.0% and 0.5% in the telemedicine and in-person consultation groups, respectively (odds ratio, 2.61; 95% confidence interval, 1.64-4.14; p < 0.001).

**Conclusions:**

Initial telemedicine consultation was significantly associated with an increase in hospitalization when compared with initial in-person consultation.

## Introduction

In Japan, initial telemedicine consultation was prohibited until March 2020. However, it was temporarily and exceptionally practiced from April 10, 2020, given the increasing difficulty in accessing medical institutions due to the coronavirus disease 2019 (COVID-19) pandemic ^(1), (2)^. Thereafter, the Ministry of Health, Labor, and Welfare (MHLW), Japan, presented guidelines for initial telemedicine consultation in January 2022. According to the guidelines, initial telemedicine consultation must, in principle, be practiced by a family doctor. However, physicians other than family doctors can conduct an initial telemedicine consultation when they can sufficiently assess the patient’s medical information and determine that telemedicine is appropriate for the patient’s symptoms ^(3)^. Subsequently, initial consultation using telecommunications devices was permanently allowed in 2022 ^(2), (4)^.

To practice telemedicine, medical institutions need to issue a notification to the MHLW. In April 2020, a total of 10,812 medical institutions submitted notifications, and this number reached 16,843 in April 2021. Furthermore, the number of medical institutions practicing initial telemedicine consultations was 4,378 in April 2020 and 7,156 in April 2021 ^(5)^. In May 2022, COVID-19 accounted for 56.5% of the total number of diseases diagnosed during initial telemedicine consultations, whereas acute upper respiratory tract infection, acute bronchitis, allergic rhinitis, and acute pharyngitis accounted for 19.8%, 16.4%, 10.1%, and 6.5%, respectively ^(6)^.

Nevertheless, telemedicine consultations are associated with certain issues, such as examinations and technical limitations, when compared with in-person consultations. According to a survey by the MHLW in 2022, 65.2% of all respondents and 20.5% of all outpatients who had received telemedicine consultations believed that they did not receive adequate examinations through telemedicine when compared with in-person consultations ^(7)^. Although telemedicine can provide an alternative to in-person consultation in primary care ^(8)^, no study has compared initial telemedicine consultation with initial in-person consultation in terms of patient outcomes such as hospitalization after outpatient management.

Therefore, we conducted a retrospective cohort study using a large administrative database to compare the proportion of hospitalizations after outpatient management in the initial telemedicine consultation and in-person consultation in patients with respiratory or digestive diseases.

## Materials and Methods

This study was approved by the institutional review board of the University of Tokyo (approval number: 2021010NI; April 23, 2021). Given that the data were anonymized, the requirement for written consent was waived.

### Data source

We used the DeSC database (DeSC Healthcare Inc.), which comprises commercially available administrative claims and health checkup data for the period between April 2014 and November 2022. This database includes information for approximately 12,500,000 individuals and health insurance claims data from three types of health insurers: (1) Health Insurance for employees of large companies, (2) the National Health Insurance for the unemployed and individual proprietors, and (3) the Advanced Elderly Medical Service System for individuals aged ≥75 years. Individual-level claims data were stored anonymously. The age distribution of individuals included in the database is similar to that of Japanese population estimates ^(9)^. The database includes the following information: a unique identifier, birth month, sex, diagnosis as encoded as per the International Classification of Diseases, 10th Revision (ICD-10) codes, procedures, and drugs dispensed on the basis of the Anatomical Therapeutic Chemical (ATC) Classification System ^(10), (11)^.

### Study design and participant selection

This retrospective cohort study used data collected between April 2020 and November 2022. We included patients who underwent an initial consultation for respiratory or digestive diseases during their outpatient visit. The index date for each patient was defined as the day the patient was diagnosed with respiratory or digestive diseases at the initial consultation. In the case of more than one initial consultation for respiratory or digestive diseases during the observation period, all episodes were included. Respiratory and digestive diseases were defined according to the ICD-10 codes (Supplementary Table 1). We focused on acute conditions because it is difficult to diagnose common chronic respiratory diseases such as asthma and chronic obstructive pulmonary disease without physical examinations or other tests during the initial telemedicine consultation ^(12)^. We excluded the following patients: (1) those whose window period was less than one year; (2) those who were diagnosed with COVID-19 (U071, U072); and (3) those who underwent other examinations from the initial consultation to the occurrence of the outcomes.

### Exposure and control groups

The exposure group comprised patients who underwent an initial telemedicine consultation through the telephone and other telecommunications devices. The control group comprised patients who underwent an initial in-person consultation.

### Outcomes

The primary outcome was hospitalization within 1 month of the index date, and the secondary outcome was death within 1 month of the index date.

### Variables

Baseline characteristics included age, sex, Charlson Comorbidity Index (CCI), medications prescribed in the year before the index date, duration of hospitalization in the year before the index date, and disease (respiratory and digestive diseases) diagnosed at the initial consultation. The CCI was calculated using the algorithm by Quan et al. ^(13)^ and categorized into five groups: 0, 1, 2, 3, 4, or >4. In addition, medications prescribed in the year before the index date were categorized into 14 groups according to the ATC codes listed in Supplementary Table 2.

### Statistical analysis

First, we performed propensity score matching to adjust for measured confounders and balance the backgrounds of the exposed and control groups. The covariates used for matching were age, sex, month, and year of the index date, CCI, each CCI component in the year before the index date, drugs prescribed in the year before the index date, the disease diagnosed on the index date, and the number of days for which the patient was hospitalized in the year before the index date. Continuous variables were age, CCI, and the number of days of hospitalization in the year before the index date; the rest were categorical variables. Logistic regression was used to estimate the propensity score, with these variables as independent variables and the implementation of telemedicine at initial consultation as the dependent variable. Matching was performed using 1:4 nearest-neighbor matching with non-reciprocal sampling. Matching was not performed if the difference in the propensity score between the exposure and control groups was more than 0.2 × the standard deviation of the propensity score. Moreover, the patient backgrounds before and after propensity score matching were compared in the exposure and control groups. Standardized differences were used to assess the balance of patient backgrounds between the two groups. A standardized difference of less than 0.1 was considered a negligible imbalance between the groups ^(14)^. The chi-square test and Fisher’s exact test were performed to compare the occurrence of outcomes in the two matched groups.

Subsequently, we applied a multilevel analysis framework to estimate the effects of variables measured at the subject and cluster levels ^(15), (16), (17)^. We analyzed the primary outcome using a multilevel logistic regression analysis with patients at the subject level and medical institutions at the cluster level. To estimate general contextual effects, we calculated the intraclass correlation coefficient (ICC). An ICC of 0% indicated no cluster effect, whereas values of 100% indicated that the cluster determined the outcome ^(18)^.

Statistical significance was set at P < 0.05. All analyses were performed using Stata/SE software (version 17.0; StataCorp, College Station, TX, USA). Statistical significance was defined as P < 0.05.

### Sensitivity analysis

We tested the robustness of our findings. First, we added COVID-19 cases (U071 and U072) to the diseases diagnosed at the initial consultation, which were prevalent on the index date. Second, we excluded patients who were hospitalized within 7 days of the index date, given they probably had a high need for hospitalization at their initial consultation.

### Subgroup analysis

First, we analyzed patients with individual diseases, including respiratory and digestive diseases. Second, we stratified patients younger than 15 years, aged 16-64 years, and older than 65 years. Third, we restricted patients to those who were hospitalized in the year before the index date. Subsequently, we conducted analyses similar to the primary analysis.

## Results

### Primary analysis

**Figure 1.**
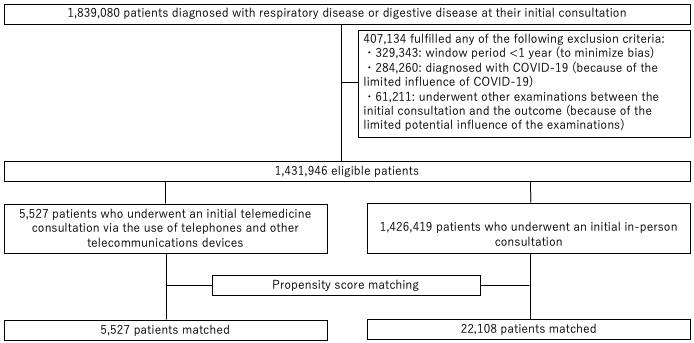
Flow diagram illustrating patient selection.

In total, this study included 1,431,946 patients. Of these, 1,426,419 and 5,527 were assigned to the control and exposure groups, respectively, with the exposure group representing approximately 0.4% of the total population. After propensity score matching, 22,108 patients and 5,527 patients were assigned to the control and exposure groups, respectively (Figure 1).

Patient backgrounds of the control and exposure groups before and after matching are listed in Table 1. The standardized differences for all covariates after propensity score matching were less than 0.1. After matching, approximately 37% of the control group and 29% of the exposure group were younger than 15 years, whereas approximately 24% of the control group and 18% of the exposure group were older than 65 years. The proportions of patients with respiratory and digestive diseases were 95% and 5%, respectively. In both groups, 36%-38% had no comorbidities, and approximately 90% had no hospitalization in the year before the index date.

**Table 1.** Baseline Characteristics of Patients.

	Overall cohort			Matched cohort		
	Control n = 1,426,419	Exposure n = 5,527	Std. diff.	Control n = 22,108	Exposure n = 5,527	Std. diff.
Age, years, n (%)			0.35			-0.03
0-15	346,359 (24.3)	1,582 (28.6)		8,185 (37.0)	1,582 (28.6)	
16-64	522,899 (36.7)	2,943 (53.3)		8,591 (38.9)	2,943 (53.3)	
≥65	557,161 (39.1)	1,002 (18.1)		5,332 (24.1)	1,002 (18.1)	
Sex, n (%)			0.05			-0.01
Male	617,999 (43.3)	2,541 (46.0)		10,235 (46.3)	2,541 (46.0)	
Female	808,420 (56.7)	2,986 (54.0)		11,873 (53.7)	2,986 (54.0)	
Observation year, n (%)			-0.36			-0.01
2020	641,655 (45.0)	1,837 (33.2)		6,652 (30.1)	1,837 (33.2)	
2021	652,451 (45.7)	2,447 (44.3)		11,325 (51.2)	2,447 (44.3)	
2022	132,313 (9.3)	1,243 (22.5)		4,131 (18.7)	1,243 (22.5)	
Disease diagnosed, n (%)						
Respiratory disease	1,193,203 (83.7)	5,251 (95.0)	-0.37	20,947 (94.8)	5,251 (95.0)	-0.01
Digestive disease	233,216 (16.4)	276 (5.0)	0.37	1,161 (5.3)	276 (5.0)	0.01
Charlson Comorbidity Index, n (%)			0.06			-0.04
0	486,919 (34.1)	1,994 (36.1)		8,496 (38.4)	1,994 (36.1)	
1	251,983 (17.7)	1,026 (18.6)		3,933 (17.8)	1,026 (18.6)	
2	193,005 (13.5)	743 (13.4)		2,880 (13.0)	743 (13.4)	
3	131,846 (9.2)	497 (9.0)		1,888 (8.5)	497 (9.0)	
≥4	362,666 (25.4)	1,267 (22.9)		4,911 (22.2)	1,267 (22.9)	
Comorbidities, n (%)						
Myocardial infarction	36,240 (2.5)	163 (3.0)	-0.03	641 (2.9)	163 (3.0)	0.00
Congestive heart failure	278,977 (19.6)	1,014 (18.4)	0.03	4,021 (18.2)	1,014 (18.4)	0.00
Peripheral vascular disease	171,963 (12.1)	638 (11.5)	0.02	2,525 (11.4)	638 (11.5)	0.00
Cerebrovascular disease	271,064 (19.0)	1,003 (18.2)	0.02	3,932 (17.8)	1,003 (18.2)	-0.01
Dementia	121,248 (8.5)	436 (7.9)	0.02	1,792 (8.1)	436 (7.9)	0.01
Chronic pulmonary disease	310,724 (21.8)	1,073 (19.4)	0.06	4,209 (19.0)	1,073 (19.4)	-0.01
Connective tissue disease	56,342 (4.0)	196 (3.6)	0.02	694 (3.1)	196 (3.6)	-0.02
Ulcer disease	223,566 (15.7)	769 (13.9)	0.05	2,968 (13.4)	769 (13.9)	-0.01
Mild liver disease	246,822 (17.3)	908 (16.4)	0.02	3,467 (15.7)	908 (16.4)	-0.02
Diabetes	40,361 (2.8)	119 (2.2)	0.04	519 (2.4)	119 (2.2)	0.01
Diabetes with end organ damage	103,555 (7.3)	342 (6.2)	0.04	1,331 (6.0)	342 (6.2)	-0.01
Hemiplegia	14,968 (1.1)	52 (0.9)	0.01	167 (0.8)	52 (0.9)	-0.02
Moderate or severe renal disease	81,852 (5.7)	290 (5.3)	0.02	1,147 (5.2)	290 (5.3)	0.00
Any tumor without metastasis, leukemia, and lymphoma	178,248 (12.5)	666 (12.1)	0.01	2,553 (11.6)	666 (12.1)	-0.02
Moderate or severe liver disease	5,748 (0.4)	11 (0.2)	0.04	39 (0.2)	11 (0.2)	-0.01
Metastatic solid tumor	26,420 (1.9)	91 (1.7)	0.02	329 (1.5)	91 (1.7)	-0.01
AIDS	516 (0.0)	3 (0.1)	-0.01	7 (0)	3 (0.1)	-0.01
Medications, n (%)						
Alimentary tract and metabolism	1,075,710 (75.4)	3,907 (70.7)	0.11	15,412 (69.7)	3,907 (70.7)	-0.02
Blood and blood-forming organs	795,148 (55.7)	2,852 (51.6)	0.08	11,164 (50.5)	2,852 (51.6)	-0.02
Cardiovascular system	1,025,093 (71.9)	3,706 (67.1)	0.10	14,702 (66.5)	3,706 (67.1)	-0.01
Dermatologicals	687,116 (48.2)	2,488 (45.0)	0.06	9,751 (44.1)	2,488 (45.0)	-0.02
Genito urinary system and sex hormones	228,059 (16.0)	755 (13.7)	0.07	2,912 (13.2)	755 (13.7)	-0.01
Systemic hormonal preparations, excl. sex hormones and insulins	355,536 (24.9)	1,198 (21.7)	0.08	4,775 (21.6)	1,198 (21.7)	0.00
Anti-infectives for systemic use	685,040 (48.0)	2,452 (44.4)	0.07	9,663 (43.7)	2,452 (44.4)	-0.01
Anti-neoplastic and immunomodulating agents	73,052 (5.1)	253 (4.6)	0.03	970 (4.4)	253 (4.6)	-0.01
Musculoskeletal system	884,790 (62.0)	3,194 (57.8)	0.09	12,632 (57.1)	3,194 (57.8)	-0.01
Nervous system	889,370 (62.4)	3,259 (59.0)	0.07	12,802 (57.9)	3,259 (59.0)	-0.02
Antiparasitic products, insecticides, and repellents	2,923 (0.2)	13 (0.2)	-0.01	50 (0.2)	13 (0.2)	0.00
Respiratory system	720,072 (50.5)	2,634 (47.7)	0.06	10,362 (46.9)	2,634 (47.7)	-0.02
Sensory organs	666,660 (46.7)	2,338 (42.3)	0.09	9,314 (42.1)	2,338 (42.3)	0.00
Various	596,311 (41.8)	2,062 (37.3)	0.09	8,178 (37.0)	2,062 (37.3)	-0.01
Duration of hospitalization days, days, n (%)			0.07			-0.02
0	1,248,609 (87.5)	4,973 (90.0)		20,056 (90.7)	4,973 (90.0)	
1~30	118,209 (8.3)	363 (6.6)		1,323 (6.0)	363 (6.6)	
31~365	59,601 (4.2)	191 (3.5)		729 (3.3)	191 (3.5)	

AIDS: acquired immunodeficiency syndrome; Std. diff.: standardized difference.

After matching, the proportion of hospitalizations within 1 month of the index date was 0.5% and 1.0% in the control and exposure groups, respectively, indicating a significant difference between the two groups (p < 0.001). The number (proportions) of deaths within 1 month of the index date was 9 (0.0%) in the control group and 1 (0.0%) in the exposure group (p = 0.429), indicating no significant difference between the two groups (Table 2).

**Table 2.** Effect Estimation before and after Propensity Score Matching.

Outcome	Before propensity matching			After propensity matching		
	Control n = 1,426,419	Exposure n = 5,527	p-Value	Control n = 22,108	Exposure n = 5,527	p-Value
Hospitalization within 1 month, n (%)	10,848 (0.8)	56 (1.0)	0.031	116 (0.5)	56 (1.0)	<0.001
Death within 1 month, n (%)	293 (0.0)	1 (0.0)	0.899	9 (0.0)	1 (0.0)	0.429

Table 3 lists the results of the multilevel logistic regression analysis. The odds ratio of telemedicine for hospitalization within 1 month of the index date was 2.61 (95% confidence interval, 1.64-4.14; p < 0.001). The general contextual effect at the patient level was small, with an ICC of 3.5%, whereas the general contextual effect of the medical institution cluster was medium, with an ICC of 63.4%.

**Table 3.** Odds Ratio of Variables for Multilevel Logistic Regression for the Outcome of Hospitalization within 1 Month of the Index Date.

	Odds ratio	95% Confidence interval	p-Value
Telemedicine (vs. in-person)	2.61	1.64-4.14	<0.001
Age	1.03	1.02-1.04	<0.001
Sex	0.88	0.60-1.29	0.521
Charlson Comorbidity Index	0.94	0.75-1.16	0.549
Comorbidities			
Myocardial infarction	0.91	0.17-4.95	0.910
Congestive heart failure	0.98	0.52-1.82	0.941
Peripheral vascular disease	0.49	0.21-1.14	0.098
Cerebrovascular disease	0.99	0.52-1.91	0.983
Dementia	1.48	0.66-3.34	0.340
Chronic pulmonary disease	1.64	0.93-2.90	0.088
Connective tissue disease	1.90	0.71-5.08	0.204
Ulcer disease	0.62	0.30-1.26	0.188
Mild liver disease	1.02	0.56-1.87	0.944
Diabetes	1.19	0.32-4.43	0.796
Diabetes with end organ damage	0.69	0.23-2.08	0.506
Hemiplegia	13.2	3.13-55.7	<0.001
Moderate or severe renal disease	1.61	0.54-4.75	0.391
Any tumor without metastasis, leukemia, and lymphoma	1.79	0.76-4.21	0.182
Moderate or severe liver disease	16.4	1.48-182.2	0.023
Metastatic solid tumor	1.08	0.12-9.79	0.943
AIDS	(omitted)		
Medications			
Alimentary tract and metabolism	1.17	0.72-1.92	0.524
Blood and blood-forming organs	0.47	0.29-0.75	0.002
Cardiovascular system	1.20	0.76-1.90	0.436
Dermatologicals	0.61	0.39-0.93	0.022
Genito urinary system and sex hormones	1.12	0.64-1.94	0.694
Systemic hormonal preparations, excl. sex hormones and insulins	1.91	1.16-3.15	0.011
Anti-infectives for systemic use	0.78	0.50-1.23	0.290
Anti-neoplastic and immunomodulating agents	0.72	0.26-2.05	0.544
Musculoskeletal system	0.75	0.49-1.16	0.197
Nervous system	0.98	0.63-1.52	0.914
Antiparasitic products, insecticides, and repellents	(omitted)		
Respiratory system	1.02	0.66-1.59	0.920
Sensory organs	1.21	0.81-1.82	0.351
Various	0.46	0.28-0.75	0.002
Disease diagnosed		
Respiratory disease	1.36	0.62-2.97	0.446
Digestive disease	Reference		
Duration of hospitalization days	1.00	1.00-1.01	0.296
Propensity score	<0.001	<0.001 to <0.001	0.001

AIDS: acquired immunodeficiency syndrome.

The sensitivity analysis results were similar to those of the primary analysis (Supplementary Table 3).

### Subgroup analysis

Among patients hospitalized in the year before the index date, the proportion of hospitalizations within 1 month of the index date was 0.7% in the control group and 0.4% in the exposure group (P = 0.344). For patients without hospitalization in the year before the index date, the proportion of hospitalizations within 1 month of the index date was 0.5% in the control group and 1.1% in the exposure group (P < 0.001) (Supplementary Table 4).

In addition, we identified 347,941 eligible patients aged <15 years; 525,842 eligible patients aged 16-64 years; and 558,163 eligible patients aged >65 years. After matching for patients aged < 15 years, hospitalization within 1 month of the index date was 0.0% in the control group and 0.0% in the exposure group (p = 0.660). For patients aged 16-64 years, hospitalization within 1 month of the index date was 0.5% in the control group and 1.1% in the exposure group (p < 0.001). For patients older than 65 years, hospitalization within 1 month of the index date was 1.4% in the control group and 2.3% in the exposure group (P = 0.028) (Supplementary Table 4).

## Discussion

Using the DeSC database, we examined the impact of initial telemedicine consultation on hospitalization when compared with in-person consultation. The occurrence of death within 1 month of the index date was rare. The results of this study revealed that initial telemedicine consultation was significantly associated with an increase in hospitalization when compared with initial in-person consultation. In addition, the results of the subgroup analyses revealed that for patients older than 16 years, initial telemedicine consultation was significantly associated with an increase in hospitalization when compared with in-person consultation. This study lacked sufficient statistical power to meaningfully compare mortality in the control and exposure groups because the number of deaths was extremely low.

Herein, we found that initial telemedicine consultation was associated with an increase in hospitalization when compared with initial in-person consultation for patients older than 16 years. This finding was similar to that of a previous study ^(19)^, which reported a higher adjusted 7-day hospitalization rate for telemedicine consultation than for in-person consultation in patients older than 18 years. These results may be due to the differences in the information available during the initial consultations. The general process of consultation includes interviews; physical examinations, including vital signs; and tests for screening, including basic tests and tests for definitive diagnosis ^(20), (21)^. The basic test in primary care is effective ^(22), (23)^. The primary difference between in-person and telemedicine consultation is the ability to perform direct physical examinations and tests. Although both in-person and telemedicine consultations allow patient interviews, physical examinations such as palpation, percussion, and auscultation are only available for in-person consultation. The importance of physical examinations, tests, and confirmation of vital signs has been reported previously ^(24), (25), (26), (27), (28)^. Considering patients older than 65 years, initial telemedicine consultation was associated with an increase in hospitalization when compared with the initial in-person consultation. This finding was similar to that of a previous study ^(29)^, which revealed that regular telemedicine consultations were associated with a higher proportion of hospitalizations within 12 months than were regular in-person consultations for patients older than 60 years. Older adults have less experience with new technologies, along with considerable sensory, memory, and other aging-related issues regarding telemedicine ^(30), (31)^. To practice appropriate consultation, older adults with these issues and multiple comorbidities ^(32)^ may require in-person consultation rather than telemedicine consultation. Our results suggest that caution should be exercised when examining patients during initial telemedicine consultations.

This study has some limitations that should be considered when interpreting the results. First, we could not comprehend the exact differences in information, including test and physical examination results, between in-person and telemedicine consultations. Second, no tests were reimbursed for telemedicine in Japan, although some tests can still be performed in telemedicine ^(33), (34)^. Third, it is difficult for older adults to use telemedicine equipment, and we could not determine whether they were able to secure an appropriate environment for telemedicine ^(35), (36)^. Fourth, the proportion of medical institutions that practiced initial telemedicine consultation was 0.72% in April 2020 and 0.48% in April 2021 ^(37)^. The proportion of patients who received an initial telemedicine consultation in this study was similar; however, we could not distinguish between telemedicine using a telephone and telemedicine using information and telecommunications devices. Because the clinical capabilities of these modalities may differ, differences in clinical outcomes could occur ^(38)^. Therefore, the results of this study have limited generalizability and should be interpreted with caution. Fifth, this study did not assess the effectiveness of telemedicine for chronic diseases; therefore, these findings may have limited generalizability to such populations. Finally, self-selection may have been a critical unmeasured confounding factor. Patients who perceived themselves as more severely ill may have preferred in-person consultations to telemedicine consultations ^(39), (40)^. Therefore, self-selection may have generated overrepresentation of healthier patients in the telemedicine group and overestimation of the effect observed in this study.

## Article Information

### Author Contributions

Takahito Morita: Conceptualization, Data curation, Formal analysis, Methods, Writing - original draft. Yusuke Sasabuchi: Formal analysis, Supervision, Writing - review and editing. Hideo Yasunaga: Funding acquisition, Supervision, Writing - review and editing.

### Conflicts of Interest

Takahito Morita was affiliated with the Ministry of Health, Labor, and Welfare in Japan at the time of submission. Yusuke Sasabuchi receives research funding from DeSC Healthcare Inc.

### IRB Approval Code and Name of the Institution

This study was approved by the institutional review board of the University of Tokyo (approval number: 2021010NI, April 23, 2021).

## Supplement

Supplementary Material
