# Supplementary material for: Hospitalization after Initial Telemedicine Versus in-Person Consultation for Outpatients with Respiratory or Digestive Diseases: A Retrospective Cohort Study
Source: JMA J. 2025 Nov 21;9(1):89–96. doi: 10.31662/jmaj.2025-0245 (PMC12889127; doi:10.31662/jmaj.2025-0245)
Supplement: Supplementary Material [file 2433-3298-9-1-0089-s001.pdf]

Supplementary materials

Supplementary Table 1. Definitions of Diseases

**Disease**	**ICD-10 code**
Respiratory disease	J00, J01, J02, J03, J04, J05, J06, J20, J21, J22
Digestive disease	K291, K297, K52, K580, K591

Supplementary Table 2. Definitions of medications in the year prior to the index date

Category	ATC code
Alimentary Tract and Metabolism	A
Blood and Blood Forming Organs	B
Cardiovascular System	C
Dermatologicals	D
Genito Urinary System and Sex Hormones	G
Systemic Hormonal Preparations, Excl. Sex Hormones and Insulins	H
Anti-infectives for Systemic Use	J
Anti-neoplastic and Immunomodulating Agents	L
Musculoskeletal System	M
Nervous System	N
Antiparasitic Products, Insecticides and Repellents	P
Respiratory System	R
Sensory Organs	S
Various	V

Supplementary Table 3. Proportion of hospitalizations within 1 month in the propensity score-matched cohort

	Control, n(%)	Exposure, n(%)	P-value
Including patients diagnosed with COVID-19 at initial consultation	574 (1.5)	166 (1.7)	0.096
Excluding patients who were hospitalized within 7 days from the index date	41 (0.2)	16 (0.3)	0.127

Supplementary Table 4. Subgroup analysis of the proportion of hospitalizations within 1 month in the propensity score-matched cohort

	Control, n (%)	Exposure, n (%)	P-value
Hospitalization within the past year from the index date			
Yes	16 (0.7)	2 (0.4)	0.344
No	100 (0.5)	54 (1.1)	< 0.001
Age			
≤15 years	1 (0.0)	0 (0.0)	0.660
16 to 64 years	42 (0.5)	33 (1.1)	< 0.001
over 65 years	73 (1.4)	23 (2.3)	0.028
